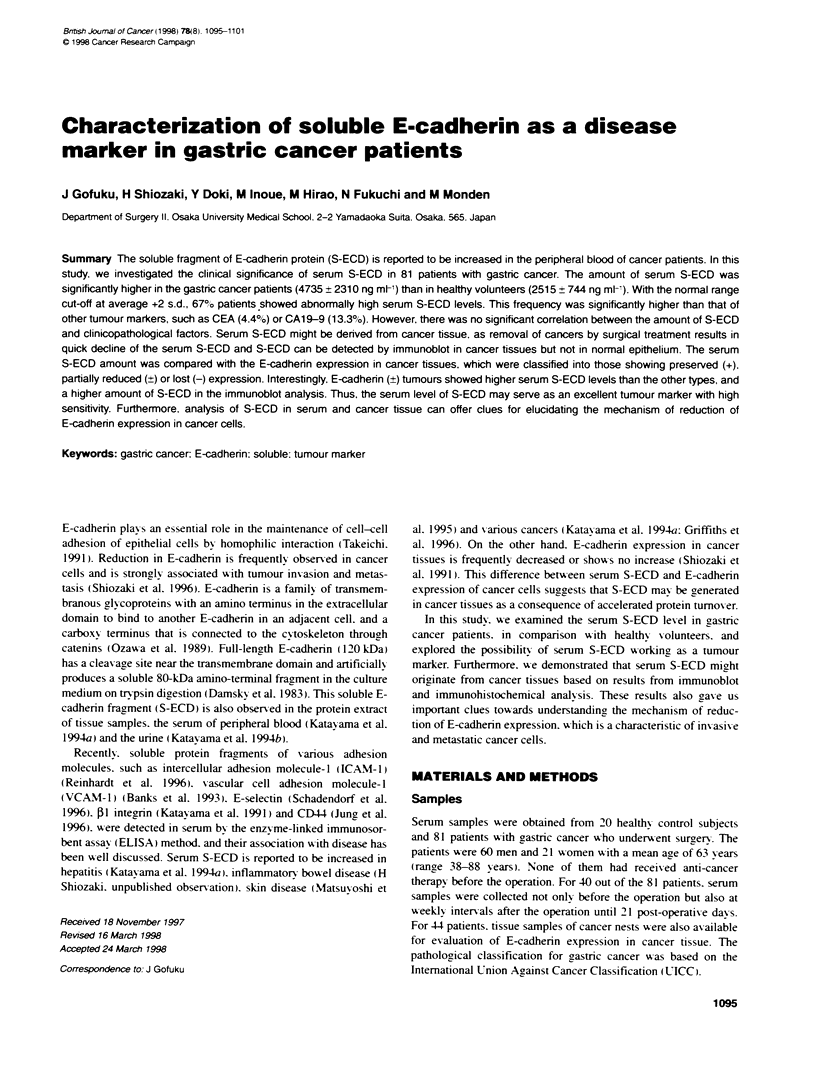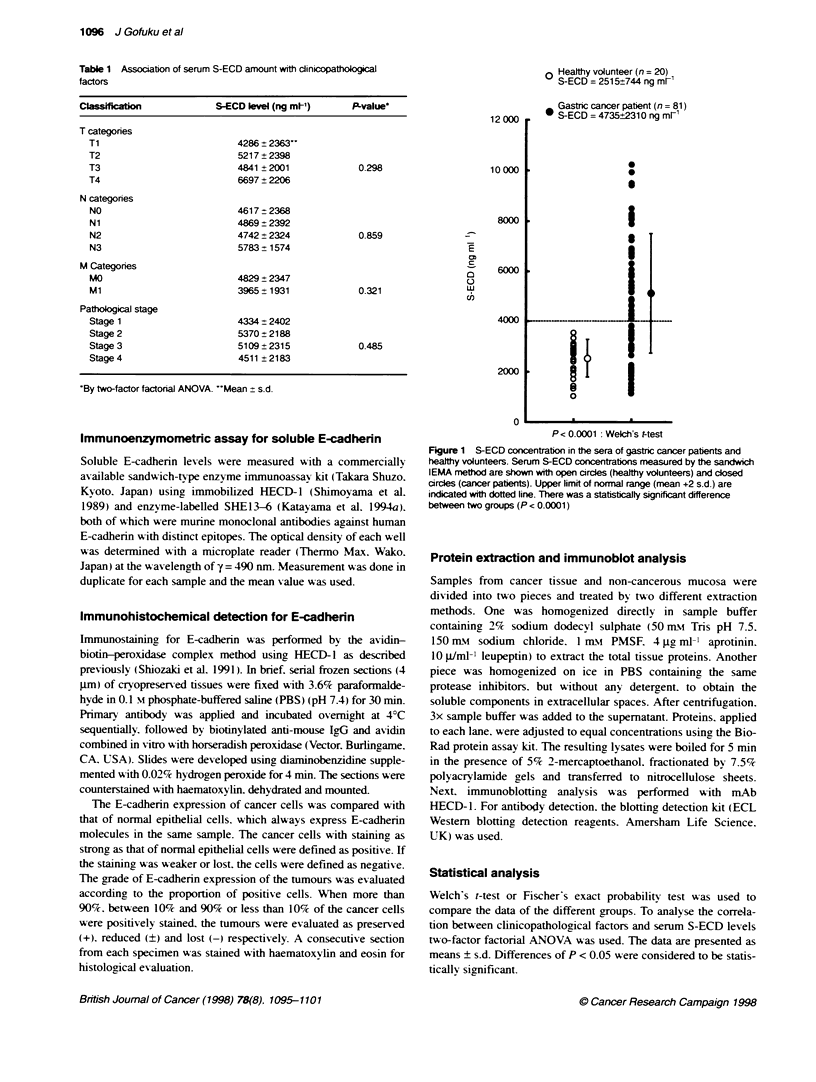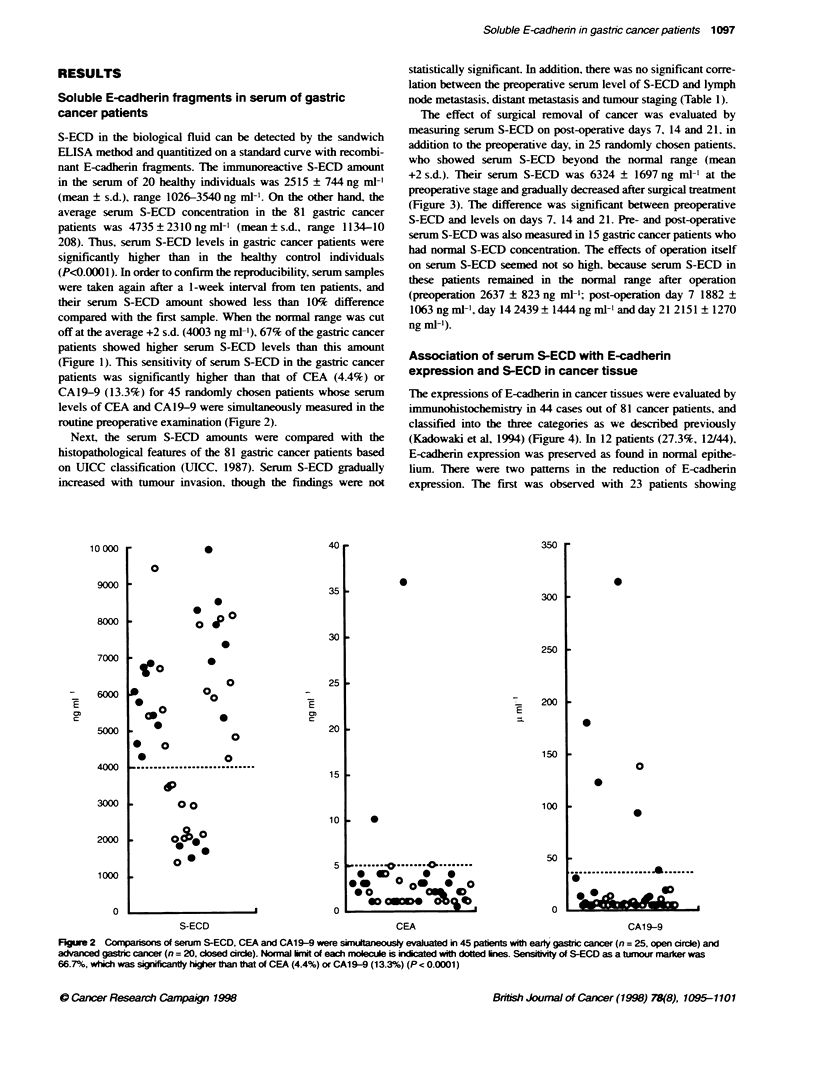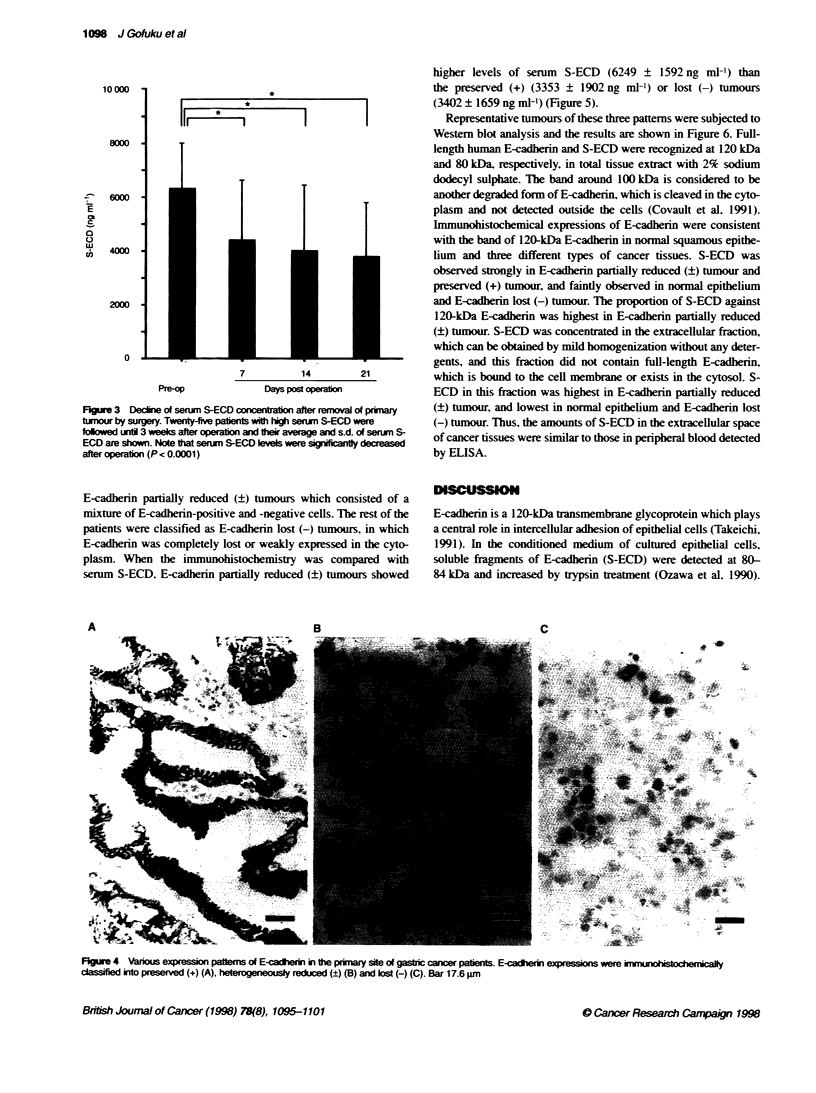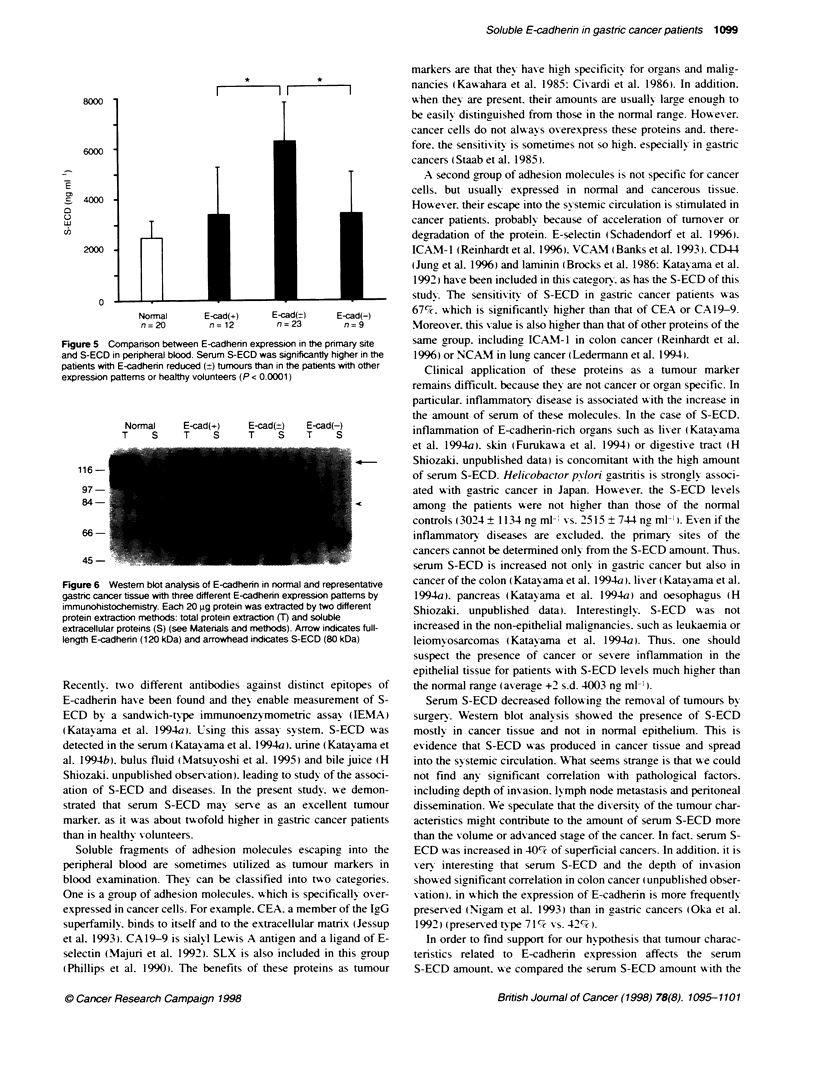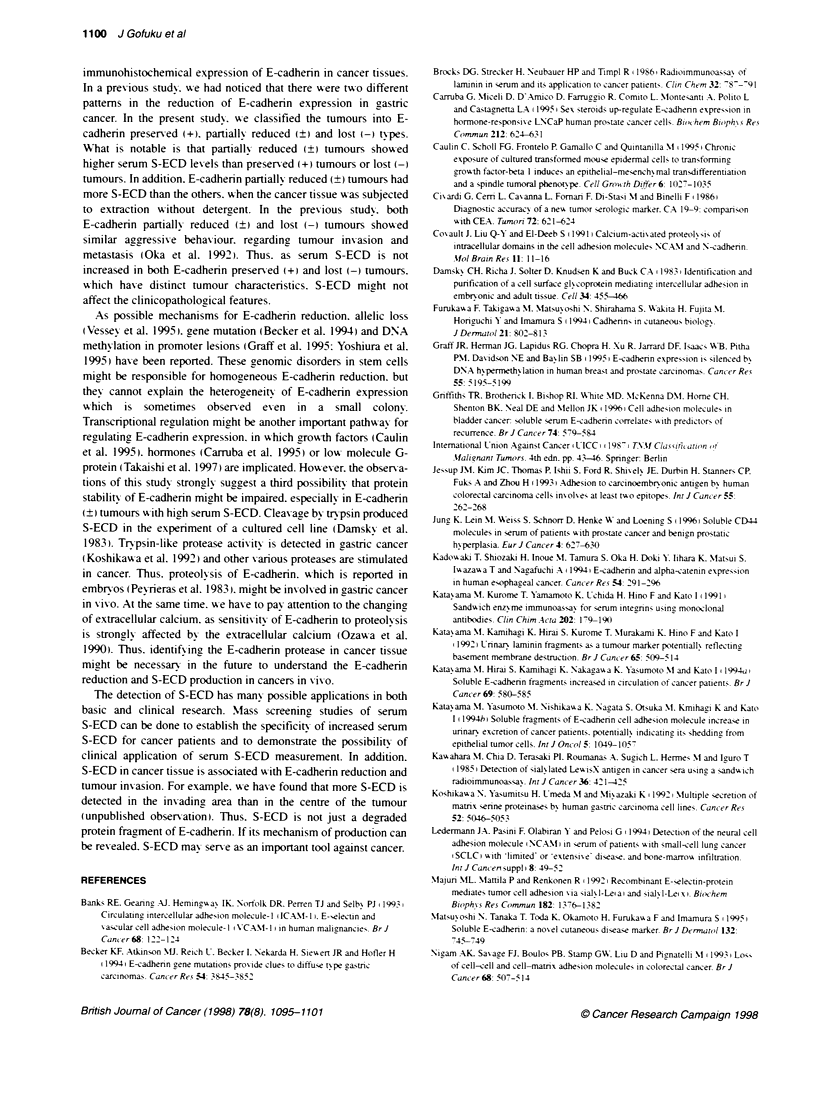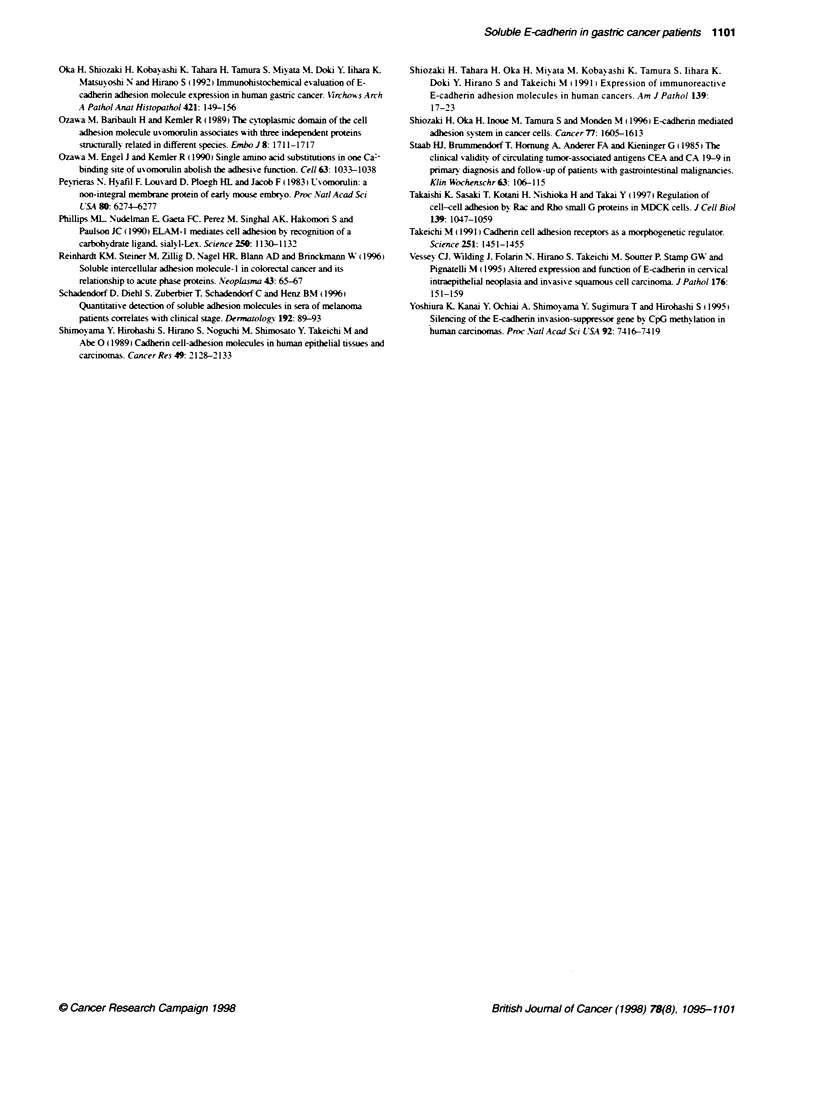# Characterization of soluble E-cadherin as a disease marker in gastric cancer patients.

**DOI:** 10.1038/bjc.1998.634

**Published:** 1998-10

**Authors:** J. Gofuku, H. Shiozaki, Y. Doki, M. Inoue, M. Hirao, N. Fukuchi, M. Monden

**Affiliations:** Department of Surgery II, Osaka University Medical School, Suita, Japan.

## Abstract

**Images:**


British Journal of Cancer (1998) 78(8), 1095–1101
© 1998 Cancer Research Campaign

### Characterization of soluble E-cadherin as a disease marker in gastric cancer patients

**J Gofuku, H Shiozaki, Y Doki, M Inoue, M Hirao, N Fukuchi and M Monden**

Department of Surgery II, Osaka University Medical School, 2-2 Yamadaoka Suita, Osaka, 565, Japan

**Summary** The soluble fragment of E-cadherin protein (S-ECD) is reported to be increased in the peripheral blood of cancer patients. In this study, we investigated the clinical significance of serum S-ECD in 81 patients with gastric cancer. The amount of serum S-ECD was significantly higher in the gastric cancer patients (4735 ± 2310 ng $ml^{-1}$) than in healthy volunteers (2515 ± 744 ng $ml^{-1}$). With the normal range cut-off at average +2 s.d., 67% patients showed abnormally high serum S-ECD levels. This frequency was significantly higher than that of other tumour markers, such as CEA (4.4%) or CA19–9 (13.3%). However, there was no significant correlation between the amount of S-ECD and clinicopathological factors. Serum S-ECD might be derived from cancer tissue, as removal of cancers by surgical treatment results in quick decline of the serum S-ECD and S-ECD can be detected by immunoblot in cancer tissues but not in normal epithelium. The serum S-ECD amount was compared with the E-cadherin expression in cancer tissues, which were classified into those showing preserved (+), partially reduced (±) or lost (–) expression. Interestingly, E-cadherin (±) tumours showed higher serum S-ECD levels than the other types, and a higher amount of S-ECD in the immunoblot analysis. Thus, the serum level of S-ECD may serve as an excellent tumour marker with high sensitivity. Furthermore, analysis of S-ECD in serum and cancer tissue can offer clues for elucidating the mechanism of reduction of E-cadherin expression in cancer cells.

**Keywords:** gastric cancer; E-cadherin; soluble; tumour marker

E-cadherin plays an essential role in the maintenance of cell–cell adhesion of epithelial cells by homophilic interaction (Takeichi, 1991). Reduction in E-cadherin is frequently observed in cancer cells and is strongly associated with tumour invasion and metastasis (Shiozaki et al, 1996). E-cadherin is a family of transmembranous glycoproteins with an amino terminus in the extracellular domain to bind to another E-cadherin in an adjacent cell, and a carboxy terminus that is connected to the cytoskeleton through catenins (Ozawa et al, 1989). Full-length E-cadherin (120 kDa) has a cleavage site near the transmembrane domain and artificially produces a soluble 80-kDa amino-terminal fragment in the culture medium on trypsin digestion (Damsky et al, 1983). This soluble E-cadherin fragment (S-ECD) is also observed in the protein extract of tissue samples, the serum of peripheral blood (Katayama et al, 1994*a*) and the urine (Katayama et al, 1994*b*).

Recently, soluble protein fragments of various adhesion molecules, such as intercellular adhesion molecule-1 (ICAM-1) (Reinhardt et al, 1996), vascular cell adhesion molecule-1 (VCAM-1) (Banks et al, 1993), E-selectin (Schadendorf et al, 1996), β1 integrin (Katayama et al, 1991) and CD44 (Jung et al, 1996), were detected in serum by the enzyme-linked immunosorbent assay (ELISA) method, and their association with disease has been well discussed. Serum S-ECD is reported to be increased in hepatitis (Katayama et al, 1994*a*), inflammatory bowel disease (H Shiozaki, unpublished observation), skin disease (Matsuyoshi et al, 1995) and various cancers (Katayama et al, 1994*a*; Griffiths et al, 1996). On the other hand, E-cadherin expression in cancer tissues is frequently decreased or shows no increase (Shiozaki et al, 1991). This difference between serum S-ECD and E-cadherin expression of cancer cells suggests that S-ECD may be generated in cancer tissues as a consequence of accelerated protein turnover.

In this study, we examined the serum S-ECD level in gastric cancer patients, in comparison with healthy volunteers, and explored the possibility of serum S-ECD working as a tumour marker. Furthermore, we demonstrated that serum S-ECD might originate from cancer tissues based on results from immunoblot and immunohistochemical analysis. These results also gave us important clues towards understanding the mechanism of reduction of E-cadherin expression, which is a characteristic of invasive and metastatic cancer cells.

*Received 18 November 1997*
*Revised 16 March 1998*
*Accepted 24 March 1998*

*Correspondence to:* J Gofuku

### MATERIALS AND METHODS

### Samples

Serum samples were obtained from 20 healthy control subjects and 81 patients with gastric cancer who underwent surgery. The patients were 60 men and 21 women with a mean age of 63 years (range 38–88 years). None of them had received anti-cancer therapy before the operation. For 40 out of the 81 patients, serum samples were collected not only before the operation but also at weekly intervals after the operation until 21 post-operative days. For 44 patients, tissue samples of cancer nests were also available for evaluation of E-cadherin expression in cancer tissue. The pathological classification for gastric cancer was based on the International Union Against Cancer Classification (UICC).

**Table 1** Association of serum S-ECD amount with clinicopathological factors

| Classification | S-ECD level (ng ml$^{-1}$) | P-value* |
|---|---|---|
| T categories | | |
| T1 | 4286 ± 2363** | |
| T2 | 5217 ± 2398 | |
| T3 | 4841 ± 2001 | 0.298 |
| T4 | 6697 ± 2206 | |
| N categories | | |
| N0 | 4617 ± 2368 | |
| N1 | 4869 ± 2392 | |
| N2 | 4742 ± 2324 | 0.859 |
| N3 | 5783 ± 1574 | |
| M Categories | | |
| M0 | 4829 ± 2347 | |
| M1 | 3965 ± 1931 | 0.321 |
| Pathological stage | | |
| Stage 1 | 4334 ± 2402 | |
| Stage 2 | 5370 ± 2188 | |
| Stage 3 | 5109 ± 2315 | 0.485 |
| Stage 4 | 4511 ± 2183 | |

*By two-factor factorial ANOVA. **Mean ± s.d.

**Figure 1** S-ECD concentration in the sera of gastric cancer patients and healthy volunteers. Serum S-ECD concentrations measured by the sandwich IEMA method are shown with open circles (healthy volunteers) and closed circles (cancer patients). Upper limit of normal range (mean +2 s.d.) are indicated with dotted line. There was a statistically significant difference between two groups ($P < 0.0001$)

### Immunoenzymometric assay for soluble E-cadherin

Soluble E-cadherin levels were measured with a commercially available sandwich-type enzyme immunoassay kit (Takara Shuzo, Kyoto, Japan) using immobilized HECD-1 (Shimoyama et al, 1989) and enzyme-labelled SHE13–6 (Katayama et al, 1994*a*), both of which were murine monoclonal antibodies against human E-cadherin with distinct epitopes. The optical density of each well was determined with a microplate reader (Thermo Max, Wako, Japan) at the wavelength of $\gamma = 490$ nm. Measurement was done in duplicate for each sample and the mean value was used.

### Immunohistochemical detection for E-cadherin

Immunostaining for E-cadherin was performed by the avidin–biotin–peroxidase complex method using HECD-1 as described previously (Shiozaki et al, 1991). In brief, serial frozen sections (4 μm) of cryopreserved tissues were fixed with 3.6% paraformaldehyde in 0.1 M phosphate-buffered saline (PBS) (pH 7.4) for 30 min. Primary antibody was applied and incubated overnight at 4°C sequentially, followed by biotinylated anti-mouse IgG and avidin combined in vitro with horseradish peroxidase (Vector, Burlingame, CA, USA). Slides were developed using diaminobenzidine supplemented with 0.02% hydrogen peroxide for 4 min. The sections were counterstained with haematoxylin, dehydrated and mounted.

The E-cadherin expression of cancer cells was compared with that of normal epithelial cells, which always express E-cadherin molecules in the same sample. The cancer cells with staining as strong as that of normal epithelial cells were defined as positive. If the staining was weaker or lost, the cells were defined as negative. The grade of E-cadherin expression of the tumours was evaluated according to the proportion of positive cells. When more than 90%, between 10% and 90% or less than 10% of the cancer cells were positively stained, the tumours were evaluated as preserved (+), reduced (±) and lost (–) respectively. A consecutive section from each specimen was stained with haematoxylin and eosin for histological evaluation.

### Protein extraction and immunoblot analysis

Samples from cancer tissue and non-cancerous mucosa were divided into two pieces and treated by two different extraction methods. One was homogenized directly in sample buffer containing 2% sodium dodecyl sulphate (50 mM Tris pH 7.5, 150 mM sodium chloride, 1 mM PMSF, 4 μg ml$^{-1}$ aprotinin, 10 μl/ml$^{-1}$ leupeptin) to extract the total tissue proteins. Another piece was homogenized on ice in PBS containing the same protease inhibitors, but without any detergent, to obtain the soluble components in extracellular spaces. After centrifugation, 3× sample buffer was added to the supernatant. Proteins, applied to each lane, were adjusted to equal concentrations using the Bio-Rad protein assay kit. The resulting lysates were boiled for 5 min in the presence of 5% 2-mercaptoethanol, fractionated by 7.5% polyacrylamide gels and transferred to nitrocellulose sheets. Next, immunoblotting analysis was performed with mAb HECD-1. For antibody detection, the blotting detection kit (ECL Western blotting detection reagents, Amersham Life Science, UK) was used.

### Statistical analysis

Welch's *t*-test or Fischer's exact probability test was used to compare the data of the different groups. To analyse the correlation between clinicopathological factors and serum S-ECD levels two-factor factorial ANOVA was used. The data are presented as means ± s.d. Differences of $P < 0.05$ were considered to be statistically significant.

© Cancer Research Campaign 1998

### RESULTS

### Soluble E-cadherin fragments in serum of gastric cancer patients

S-ECD in the biological fluid can be detected by the sandwich ELISA method and quantitized on a standard curve with recombinant E-cadherin fragments. The immunoreactive S-ECD amount in the serum of 20 healthy individuals was 2515 ± 744 ng $ml^{-1}$ (mean ± s.d.), range 1026–3540 ng $ml^{-1}$. On the other hand, the average serum S-ECD concentration in the 81 gastric cancer patients was 4735 ± 2310 ng $ml^{-1}$ (mean ± s.d., range 1134–10 208). Thus, serum S-ECD levels in gastric cancer patients were significantly higher than in the healthy control individuals ($P$<0.0001). In order to confirm the reproducibility, serum samples were taken again after a 1-week interval from ten patients, and their serum S-ECD amount showed less than 10% difference compared with the first sample. When the normal range was cut off at the average +2 s.d. (4003 ng $ml^{-1}$), 67% of the gastric cancer patients showed higher serum S-ECD levels than this amount (Figure 1). This sensitivity of serum S-ECD in the gastric cancer patients was significantly higher than that of CEA (4.4%) or CA19–9 (13.3%) for 45 randomly chosen patients whose serum levels of CEA and CA19–9 were simultaneously measured in the routine preoperative examination (Figure 2).

Next, the serum S-ECD amounts were compared with the histopathological features of the 81 gastric cancer patients based on UICC classification (UICC, 1987). Serum S-ECD gradually increased with tumour invasion, though the findings were not statistically significant. In addition, there was no significant correlation between the preoperative serum level of S-ECD and lymph node metastasis, distant metastasis and tumour staging (Table 1).

The effect of surgical removal of cancer was evaluated by measuring serum S-ECD on post-operative days 7, 14 and 21, in addition to the preoperative day, in 25 randomly chosen patients, who showed serum S-ECD beyond the normal range (mean +2 s.d.). Their serum S-ECD was 6324 ± 1697 ng $ml^{-1}$ at the preoperative stage and gradually decreased after surgical treatment (Figure 3). The difference was significant between preoperative S-ECD and levels on days 7, 14 and 21. Pre- and post-operative serum S-ECD was also measured in 15 gastric cancer patients who had normal S-ECD concentration. The effects of operation itself on serum S-ECD seemed not so high, because serum S-ECD in these patients remained in the normal range after operation (preoperation 2637 ± 823 ng $ml^{-1}$; post-operation day 7 1882 ± 1063 ng $ml^{-1}$, day 14 2439 ± 1444 ng $ml^{-1}$ and day 21 2151 ± 1270 ng $ml^{-1}$).

### Association of serum S-ECD with E-cadherin expression and S-ECD in cancer tissue

The expressions of E-cadherin in cancer tissues were evaluated by immunohistochemistry in 44 cases out of 81 cancer patients, and classified into the three categories as we described previously (Kadowaki et al, 1994) (Figure 4). In 12 patients (27.3%, 12/44), E-cadherin expression was preserved as found in normal epithelium. There were two patterns in the reduction of E-cadherin expression. The first was observed with 23 patients showing

**Figure 2** Comparisons of serum S-ECD, CEA and CA19–9 were simultaneously evaluated in 45 patients with early gastric cancer ($n$ = 25, open circle) and advanced gastric cancer ($n$ = 20, closed circle). Normal limit of each molecule is indicated with dotted lines. Sensitivity of S-ECD as a tumour marker was 66.7%, which was significantly higher than that of CEA (4.4%) or CA19–9 (13.3%) ($P$ < 0.0001)

**Figure 3** Decline of serum S-ECD concentration after removal of primary tumour by surgery. Twenty-five patients with high serum S-ECD were followed until 3 weeks after operation and their average and s.d. of serum S-ECD are shown. Note that serum S-ECD levels were significantly decreased after operation ($P < 0.0001$)

E-cadherin partially reduced (±) tumours which consisted of a mixture of E-cadherin-positive and -negative cells. The rest of the patients were classified as E-cadherin lost (–) tumours, in which E-cadherin was completely lost or weakly expressed in the cytoplasm. When the immunohistochemistry was compared with serum S-ECD, E-cadherin partially reduced (±) tumours showed higher levels of serum S-ECD (6249 ± 1592 ng ml$^{-1}$) than the preserved (+) (3353 ± 1902 ng ml$^{-1}$) or lost (–) tumours (3402 ± 1659 ng ml$^{-1}$) (Figure 5).

Representative tumours of these three patterns were subjected to Western blot analysis and the results are shown in Figure 6. Full-length human E-cadherin and S-ECD were recognized at 120 kDa and 80 kDa, respectively, in total tissue extract with 2% sodium dodecyl sulphate. The band around 100 kDa is considered to be another degraded form of E-cadherin, which is cleaved in the cytoplasm and not detected outside the cells (Covault et al, 1991). Immunohistochemical expressions of E-cadherin were consistent with the band of 120-kDa E-cadherin in normal squamous epithelium and three different types of cancer tissues. S-ECD was observed strongly in E-cadherin partially reduced (±) tumour and preserved (+) tumour, and faintly observed in normal epithelium and E-cadherin lost (–) tumour. The proportion of S-ECD against 120-kDa E-cadherin was highest in E-cadherin partially reduced (±) tumour. S-ECD was concentrated in the extracellular fraction, which can be obtained by mild homogenization without any detergents, and this fraction did not contain full-length E-cadherin, which is bound to the cell membrane or exists in the cytosol. S-ECD in this fraction was highest in E-cadherin partially reduced (±) tumour, and lowest in normal epithelium and E-cadherin lost (–) tumour. Thus, the amounts of S-ECD in the extracellular space of cancer tissues were similar to those in peripheral blood detected by ELISA.

### DISCUSSION

E-cadherin is a 120-kDa transmembrane glycoprotein which plays a central role in intercellular adhesion of epithelial cells (Takeichi, 1991). In the conditioned medium of cultured epithelial cells, soluble fragments of E-cadherin (S-ECD) were detected at 80–84 kDa and increased by trypsin treatment (Ozawa et al, 1990).

**Figure 4** Various expression patterns of E-cadherin in the primary site of gastric cancer patients. E-cadherin expressions were immunohistochemically classified into preserved (+) (A), heterogeneously reduced (±) (B) and lost (–) (C). Bar 17.6 μm

 © Cancer Research Campaign 1998

**Figure 5** Comparison between E-cadherin expression in the primary site and S-ECD in peripheral blood. Serum S-ECD was significantly higher in the patients with E-cadherin reduced (±) tumours than in the patients with other expression patterns or healthy volunteers ($P < 0.0001$)

**Figure 6** Western blot analysis of E-cadherin in normal and representative gastric cancer tissue with three different E-cadherin expression patterns by immunohistochemistry. Each 20 µg protein was extracted by two different protein extraction methods: total protein extraction (T) and soluble extracellular proteins (S) (see Materials and methods). Arrow indicates full-length E-cadherin (120 kDa) and arrowhead indicates S-ECD (80 kDa)

Recently, two different antibodies against distinct epitopes of E-cadherin have been found and they enable measurement of S-ECD by a sandwich-type immunoenzymometric assay (IEMA) (Katayama et al. 1994*a*). Using this assay system, S-ECD was detected in the serum (Katayama et al. 1994*a*), urine (Katayama et al. 1994*b*), bulus fluid (Matsuyoshi et al. 1995) and bile juice (H Shiozaki, unpublished observation), leading to study of the association of S-ECD and diseases. In the present study, we demonstrated that serum S-ECD may serve as an excellent tumour marker, as it was about twofold higher in gastric cancer patients than in healthy volunteers.

Soluble fragments of adhesion molecules escaping into the peripheral blood are sometimes utilized as tumour markers in blood examination. They can be classified into two categories. One is a group of adhesion molecules, which is specifically overexpressed in cancer cells. For example, CEA, a member of the IgG superfamily, binds to itself and to the extracellular matrix (Jessup et al. 1993). CA19–9 is sialyl Lewis A antigen and a ligand of E-selectin (Majuri et al. 1992). SLX is also included in this group (Phillips et al. 1990). The benefits of these proteins as tumour markers are that they have high specificity for organs and malignancies (Kawahara et al. 1985; Civardi et al. 1986). In addition, when they are present, their amounts are usually large enough to be easily distinguished from those in the normal range. However, cancer cells do not always overexpress these proteins and, therefore, the sensitivity is sometimes not so high, especially in gastric cancers (Staab et al. 1985).

A second group of adhesion molecules is not specific for cancer cells, but usually expressed in normal and cancerous tissue. However, their escape into the systemic circulation is stimulated in cancer patients, probably because of acceleration of turnover or degradation of the protein. E-selectin (Schadendorf et al. 1996), ICAM-1 (Reinhardt et al. 1996), VCAM (Banks et al. 1993), CD44 (Jung et al. 1996) and laminin (Brocks et al. 1986; Katayama et al. 1992) have been included in this category, as has the S-ECD of this study. The sensitivity of S-ECD in gastric cancer patients was 67%, which is significantly higher than that of CEA or CA19–9. Moreover, this value is also higher than that of other proteins of the same group, including ICAM-1 in colon cancer (Reinhardt et al. 1996) or NCAM in lung cancer (Ledermann et al. 1994).

Clinical application of these proteins as a tumour marker remains difficult, because they are not cancer or organ specific. In particular, inflammatory disease is associated with the increase in the amount of serum of these molecules. In the case of S-ECD, inflammation of E-cadherin-rich organs such as liver (Katayama et al. 1994*a*), skin (Furukawa et al. 1994) or digestive tract (H Shiozaki, unpublished data) is concomitant with the high amount of serum S-ECD. *Helicobactor pylori* gastritis is strongly associated with gastric cancer in Japan. However, the S-ECD levels among the patients were not higher than those of the normal controls (3024 ± 1134 ng ml$^{-1}$ vs. 2515 ± 744 ng ml$^{-1}$). Even if the inflammatory diseases are excluded, the primary sites of the cancers cannot be determined only from the S-ECD amount. Thus, serum S-ECD is increased not only in gastric cancer but also in cancer of the colon (Katayama et al. 1994*a*), liver (Katayama et al. 1994*a*), pancreas (Katayama et al. 1994*a*) and oesophagus (H Shiozaki, unpublished data). Interestingly, S-ECD was not increased in the non-epithelial malignancies, such as leukaemia or leiomyosarcomas (Katayama et al. 1994*a*). Thus, one should suspect the presence of cancer or severe inflammation in the epithelial tissue for patients with S-ECD levels much higher than the normal range (average +2 s.d. 4003 ng ml$^{-1}$).

Serum S-ECD decreased following the removal of tumours by surgery. Western blot analysis showed the presence of S-ECD mostly in cancer tissue and not in normal epithelium. This is evidence that S-ECD was produced in cancer tissue and spread into the systemic circulation. What seems strange is that we could not find any significant correlation with pathological factors, including depth of invasion, lymph node metastasis and peritoneal dissemination. We speculate that the diversity of the tumour characteristics might contribute to the amount of serum S-ECD more than the volume or advanced stage of the cancer. In fact, serum S-ECD was increased in 40% of superficial cancers. In addition, it is very interesting that serum S-ECD and the depth of invasion showed significant correlation in colon cancer (unpublished observation), in which the expression of E-cadherin is more frequently preserved (Nigam et al. 1993) than in gastric cancers (Oka et al. 1992) (preserved type 71% vs. 42%).

In order to find support for our hypothesis that tumour characteristics related to E-cadherin expression affects the serum S-ECD amount, we compared the serum S-ECD amount with the immunohistochemical expression of E-cadherin in cancer tissues. In a previous study, we had noticed that there were two different patterns in the reduction of E-cadherin expression in gastric cancer. In the present study, we classified the tumours into E-cadherin preserved (+), partially reduced (±) and lost (−) types. What is notable is that partially reduced (±) tumours showed higher serum S-ECD levels than preserved (+) tumours or lost (−) tumours. In addition, E-cadherin partially reduced (±) tumours had more S-ECD than the others, when the cancer tissue was subjected to extraction without detergent. In the previous study, both E-cadherin partially reduced (±) and lost (−) tumours showed similar aggressive behaviour, regarding tumour invasion and metastasis (Oka et al, 1992). Thus, as serum S-ECD is not increased in both E-cadherin preserved (+) and lost (−) tumours, which have distinct tumour characteristics, S-ECD might not affect the clinicopathological features.

As possible mechanisms for E-cadherin reduction, allelic loss (Vessey et al, 1995), gene mutation (Becker et al, 1994) and DNA methylation in promoter lesions (Graff et al, 1995; Yoshiura et al, 1995) have been reported. These genomic disorders in stem cells might be responsible for homogeneous E-cadherin reduction, but they cannot explain the heterogeneity of E-cadherin expression which is sometimes observed even in a small colony. Transcriptional regulation might be another important pathway for regulating E-cadherin expression, in which growth factors (Caulin et al, 1995), hormones (Carruba et al, 1995) or low molecule G-protein (Takaishi et al, 1997) are implicated. However, the observations of this study strongly suggest a third possibility that protein stability of E-cadherin might be impaired, especially in E-cadherin (±) tumours with high serum S-ECD. Cleavage by trypsin produced S-ECD in the experiment of a cultured cell line (Damsky et al, 1983). Trypsin-like protease activity is detected in gastric cancer (Koshikawa et al, 1992) and other various proteases are stimulated in cancer. Thus, proteolysis of E-cadherin, which is reported in embryos (Peyrieras et al, 1983), might be involved in gastric cancer in vivo. At the same time, we have to pay attention to the changing of extracellular calcium, as sensitivity of E-cadherin to proteolysis is strongly affected by the extracellular calcium (Ozawa et al, 1990). Thus, identifying the E-cadherin protease in cancer tissue might be necessary in the future to understand the E-cadherin reduction and S-ECD production in cancers in vivo.

The detection of S-ECD has many possible applications in both basic and clinical research. Mass screening studies of serum S-ECD can be done to establish the specificity of increased serum S-ECD for cancer patients and to demonstrate the possibility of clinical application of serum S-ECD measurement. In addition, S-ECD in cancer tissue is associated with E-cadherin reduction and tumour invasion. For example, we have found that more S-ECD is detected in the invading area than in the centre of the tumour (unpublished observation). Thus, S-ECD is not just a degraded protein fragment of E-cadherin. If its mechanism of production can be revealed, S-ECD may serve as an important tool against cancer.

### REFERENCES

Banks RE, Gearing AJ, Hemingway IK, Norfolk DR, Perren TJ and Selby PJ (1993) Circulating intercellular adhesion molecule-1 (ICAM-1), E-selectin and vascular cell adhesion molecule-1 (VCAM-1) in human malignancies. *Br J Cancer* **68**: 122–124

Becker KF, Atkinson MJ, Reich U, Becker I, Nekarda H, Siewert JR and Hofler H (1994) E-cadherin gene mutations provide clues to diffuse type gastric carcinomas. *Cancer Res* **54**: 3845–3852

Brocks DG, Strecker H, Neubauer HP and Timpl R (1986) Radioimmunoassay of laminin in serum and its application to cancer patients. *Clin Chem* **32**: 787–791

Carruba G, Miceli D, D'Amico D, Farruggio R, Comito L, Montesanti A, Polito L and Castagnetta LA (1995) Sex steroids up-regulate E-cadherin expression in hormone-responsive LNCaP human prostate cancer cells. *Biochem Biophys Res Commun* **212**: 624–631

Caulin C, Scholl FG, Frontelo P, Gamallo C and Quintanilla M (1995) Chronic exposure of cultured transformed mouse epidermal cells to transforming growth factor-beta 1 induces an epithelial–mesenchymal transdifferentiation and a spindle tumoral phenotype. *Cell Growth Differ* **6**: 1027–1035

Civardi G, Cerri L, Cavanna L, Fornari F, Di-Stasi M and Binelli F (1986) Diagnostic accuracy of a new tumor serologic marker, CA 19–9: comparison with CEA. *Tumori* **72**: 621–624

Covault J, Liu Q-Y and El-Deeb S (1991) Calcium-activated proteolysis of intracellular domains in the cell adhesion molecules NCAM and N-cadherin. *Mol Brain Res* **11**: 11–16

Damsky CH, Richa J, Solter D, Knudsen K and Buck CA (1983) Identification and purification of a cell surface glycoprotein mediating intercellular adhesion in embryonic and adult tissue. *Cell* **34**: 455–466

Furukawa F, Takigawa M, Matsuyoshi N, Shirahama S, Wakita H, Fujita M, Horiguchi Y and Imamura S (1994) Cadherins in cutaneous biology. *J Dermatol* **21**: 802–813

Graff JR, Herman JG, Lapidus RG, Chopra H, Xu R, Jarrard DF, Isaacs WB, Pitha PM, Davidson NE and Baylin SB (1995) E-cadherin expression is silenced by DNA hypermethylation in human breast and prostate carcinomas. *Cancer Res* **55**: 5195–5199

Griffiths TR, Brotherick I, Bishop RI, White MD, McKenna DM, Horne CH, Shenton BK, Neal DE and Mellon JK (1996) Cell adhesion molecules in bladder cancer: soluble serum E-cadherin correlates with predictors of recurrence. *Br J Cancer* **74**: 579–584

International Union Against Cancer (UICC) (1987) *TNM Classification of Malignant Tumors*. 4th edn, pp. 43–46. Springer: Berlin

Jessup JM, Kim JC, Thomas P, Ishii S, Ford R, Shively JE, Durbin H, Stanners CP, Fuks A and Zhou H (1993) Adhesion to carcinoembryonic antigen by human colorectal carcinoma cells involves at least two epitopes. *Int J Cancer* **55**: 262–268

Jung K, Lein M, Weiss S, Schnorr D, Henke W and Loening S (1996) Soluble CD44 molecules in serum of patients with prostate cancer and benign prostatic hyperplasia. *Eur J Cancer* **4**: 627–630

Kadowaki T, Shiozaki H, Inoue M, Tamura S, Oka H, Doki Y, Iihara K, Matsui S, Iwazawa T and Nagafuchi A (1994) E-cadherin and alpha-catenin expression in human esophageal cancer. *Cancer Res* **54**: 291–296

Katayama M, Kurome T, Yamamoto K, Uchida H, Hino F and Kato I (1991) Sandwich enzyme immunoassay for serum integrins using monoclonal antibodies. *Clin Chim Acta* **202**: 179–190

Katayama M, Kamihagi K, Hirai S, Kurome T, Murakami K, Hino F and Kato I (1992) Urinary laminin fragments as a tumour marker potentially reflecting basement membrane destruction. *Br J Cancer* **65**: 509–514

Katayama M, Hirai S, Kamihagi K, Nakagawa K, Yasumoto M and Kato I (1994*a*) Soluble E-cadherin fragments increased in circulation of cancer patients. *Br J Cancer* **69**: 580–585

Katayama M, Yasumoto M, Nishikawa K, Nagata S, Otsuka M, Kmihagi K and Kato I (1994*b*) Soluble fragments of E-cadherin cell adhesion molecule increase in urinary excretion of cancer patients, potentially indicating its shedding from epithelial tumor cells. *Int J Oncol* **5**: 1049–1057

Kawahara M, Chia D, Terasaki PI, Roumanas A, Sugich L, Hermes M and Iguro T (1985) Detection of sialylated LewisX antigen in cancer sera using a sandwich radioimmunoassay. *Int J Cancer* **36**: 421–425

Koshikawa N, Yasumitsu H, Umeda M and Miyazaki K (1992) Multiple secretion of matrix serine proteinases by human gastric carcinoma cell lines. *Cancer Res* **52**: 5046–5053

Ledermann JA, Pasini F, Olabiran Y and Pelosi G (1994) Detection of the neural cell adhesion molecule (NCAM) in serum of patients with small-cell lung cancer (SCLC) with 'limited' or 'extensive' disease, and bone-marrow infiltration. *Int J Cancer* (suppl) **8**: 49–52

Majuri ML, Mattila P and Renkonen R (1992) Recombinant E-selectin-protein mediates tumor cell adhesion via sialyl-Le(a) and sialyl-Le(x). *Biochem Biophys Res Commun* **182**: 1376–1382

Matsuyoshi N, Tanaka T, Toda K, Okamoto H, Furukawa F and Imamura S (1995) Soluble E-cadherin: a novel cutaneous disease marker. *Br J Dermatol* **132**: 745–749

Nigam AK, Savage FJ, Boulos PB, Stamp GW, Liu D and Pignatelli M (1993) Loss of cell–cell and cell–matrix adhesion molecules in colorectal cancer. *Br J Cancer* **68**: 507–514

© Cancer Research Campaign 1998

Oka H, Shiozaki H, Kobayashi K, Tahara H, Tamura S, Miyata M, Doki Y, Iihara K, Matsuyoshi N and Hirano S (1992) Immunohistochemical evaluation of E-cadherin adhesion molecule expression in human gastric cancer. *Virchows Arch A Pathol Anat Histopathol* **421**: 149–156

Ozawa M, Baribault H and Kemler R (1989) The cytoplasmic domain of the cell adhesion molecule uvomorulin associates with three independent proteins structurally related in different species. *Embo J* **8**: 1711–1717

Ozawa M, Engel J and Kemler R (1990) Single amino acid substitutions in one $Ca^{2+}$ binding site of uvomorulin abolish the adhesive function. *Cell* **63**: 1033–1038

Peyrieras N, Hyafil F, Louvard D, Ploegh HL and Jacob F (1983) Uvomorulin: a non-integral membrane protein of early mouse embryo. *Proc Natl Acad Sci USA* **80**: 6274–6277

Phillips ML, Nudelman E, Gaeta FC, Perez M, Singhal AK, Hakomori S and Paulson JC (1990) ELAM-1 mediates cell adhesion by recognition of a carbohydrate ligand, sialyl-Lex. *Science* **250**: 1130–1132

Reinhardt KM, Steiner M, Zillig D, Nagel HR, Blann AD and Brinckmann W (1996) Soluble intercellular adhesion molecule-1 in colorectal cancer and its relationship to acute phase proteins. *Neoplasma* **43**: 65–67

Schadendorf D, Diehl S, Zuberbier T, Schadendorf C and Henz BM (1996) Quantitative detection of soluble adhesion molecules in sera of melanoma patients correlates with clinical stage. *Dermatology* **192**: 89–93

Shimoyama Y, Hirohashi S, Hirano S, Noguchi M, Shimosato Y, Takeichi M and Abe O (1989) Cadherin cell-adhesion molecules in human epithelial tissues and carcinomas. *Cancer Res* **49**: 2128–2133

Shiozaki H, Tahara H, Oka H, Miyata M, Kobayashi K, Tamura S, Iihara K, Doki Y, Hirano S and Takeichi M (1991) Expression of immunoreactive E-cadherin adhesion molecules in human cancers. *Am J Pathol* **139**: 17–23

Shiozaki H, Oka H, Inoue M, Tamura S and Monden M (1996) E-cadherin mediated adhesion system in cancer cells. *Cancer* **77**: 1605–1613

Staab HJ, Brummendorf T, Hornung A, Anderer FA and Kieninger G (1985) The clinical validity of circulating tumor-associated antigens CEA and CA 19–9 in primary diagnosis and follow-up of patients with gastrointestinal malignancies. *Klin Wochenschr* **63**: 106–115

Takaishi K, Sasaki T, Kotani H, Nishioka H and Takai Y (1997) Regulation of cell–cell adhesion by Rac and Rho small G proteins in MDCK cells. *J Cell Biol* **139**: 1047–1059

Takeichi M (1991) Cadherin cell adhesion receptors as a morphogenetic regulator. *Science* **251**: 1451–1455

Vessey CJ, Wilding J, Folarin N, Hirano S, Takeichi M, Soutter P, Stamp GW and Pignatelli M (1995) Altered expression and function of E-cadherin in cervical intraepithelial neoplasia and invasive squamous cell carcinoma. *J Pathol* **176**: 151–159

Yoshiura K, Kanai Y, Ochiai A, Shimoyama Y, Sugimura T and Hirohashi S (1995) Silencing of the E-cadherin invasion-suppressor gene by CpG methylation in human carcinomas. *Proc Natl Acad Sci USA* **92**: 7416–7419

© Cancer Research Campaign 1998